# TRPM7 promotes lipopolysaccharide‐induced inflammatory dysfunction in renal tubular epithelial cells

**DOI:** 10.1002/iid3.641

**Published:** 2022-06-20

**Authors:** Yan Sun, Xiaobing Chen, Yongpeng Xie, Yanli Wang, Qian Zhang, Yu Lu, Xiaomin Li

**Affiliations:** ^1^ Department of Emergency Medicine Lianyungang Clinical College of Nanjing Medical University Lianyungang China

**Keywords:** inflammation, Kruppel‐like factor 2, renal tubular epithelial cells, sepsis, TRPM7

## Abstract

**Background:**

Sepsis‐associated acute kidney injury (S‐AKI) has been reported to affect 30%–50% of all sepsis patients; this condition is associated with a notable fatality rate. Following lipopolysaccharide (LPS) stimulation, the expression of transient receptor potential cation channel subfamily M member 7 (TRPM7), a nonselective cation channel expressed by the renal tubular epithelial cells (RTECs) was found to be upregulated. We aimed to determine how TRPM7 functions in S‐AKI.

**Methods:**

To establish an in vitro model of S‐AKI, RTECs were treated with LPS. The effect of TRPM7 knockdown on cell viability, lactate dehydrogenase (LDH) release, apoptosis, inflammation, and oxidative stress was studied. The binding site between Kruppel‐like factor 2 (KLF2) and TRPM7 was predicted using JASPAR. The influence of KLF2 on the regulatory roles of TRPM7 in cells, as well as the effect of their knockdown on the MAPK signaling pathway, was investigated.

**Results:**

TRPM7 was upregulated in LPS‐treated cells, and knocking improved cell viability, reduced LDH levels, and minimized apoptosis, inflammation, and oxidative stress. KLF2 was shown to be associated with TRPM7 and its level decreased in LPS‐treated cells. KLF2 knockdown increased TRPM7 expression and reversed the effects of TRPM7 knockdown in LPS‐treated cells, including suppression of p38 MAPK, ERK1/2, and JNK activation.

**Conclusion:**

Taken together, our results show that TRPM7 is negatively regulated by KLF2 and promotes LPS‐induced inflammatory dysfunction by activating the MAPK pathway in RTECs. The theoretical foundation for the prevention and management of S‐AKI is laid out in this article.

## Graphical abstract

TRPM7 is negatively regulated by KLF2 transcription and promotes lipopolysaccharide‐induced inflammatory dysfunction via activating the MAPK pathway in renal tubular epithelial cells. This article lays the necessary theoretical basis for the prevention and treatment of sepsis‐associated acute kidney injury.

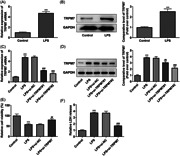

## 1 INTRODUCTION

Sepsis is a life‐threatening organ malfunction caused by an imbalance in the body's response to infection due to trauma, infection, shock, surgery, and other factors.^1^ The fatality rate of patients with sepsis has been dramatically lowered because of a better understanding of the pathophysiology of sepsis, and improvements in critical care^2^; however, sepsis is still the most common acute and critical illness in intensive care units, with a death rate of more than 20%.^3^ The pathophysiology of organ dysfunction due to sepsis is highly complex, and numerous unexplored aspects remain.^4^ Sepsis is thought to be associated with systemic inflammatory response syndrome, an imbalance of overcompensated anti‐inflammatory response, coagulation dysfunction, immunological paralysis, aberrant neuroendocrine function, and microcirculation disorders.^5^, ^6^ Acute kidney injury is the most universal organ dysfunction occurring due to sepsis.^7^ It is characterized by acute renal failure, which is majorly manifested by insufficient blood filtration, water, and ion regulatory disturbances, and abnormal urine output.^8^ The incidence of sepsis‐associated acute kidney injury is high, accounting for 30%–50% of all sepsis patients.^9^ According to previous research, the production of large amounts of inflammatory mediators, aberrant renal hemodynamics, and immunological dysfunction are all high‐risk factors for S‐AKI.^10^ Inflammatory mediators have a very major role among these risk variables, even transcending the traditional concept of acute tubular necrosis.^11^

Transient receptor potential cation channel subfamily M member 7 (TRPM7) is a nonselective cation channel that functions as both an ion channel and protein kinase in proximal renal tubular epithelial cells (RTECs).^12^ TRPM7 plays an extremely marked role in regulating intracellular and extracellular signal transmission and influences a variety of cellular physiological functions.^13^ TRPM7 is abundantly expressed in the kidney and is increased in an ischemia–reperfusion (I/R) injury. As such, silencing TRPM7 alleviates renal I/R injury parameters and renal function and inhibits inflammation and apoptosis.^14^ Moreover, lipopolysaccharide (LPS) promotes the course of sepsis by upregulating the expression of TRPM7, reducing the viability of human umbilical vein endothelial cells, inducing apoptosis, inflammatory damage, and oxidative stress.^15^ Endothelial cell permeability is notably enhanced during sepsis, leading to renal failure and patient mortality. Endotoxemia induced an increase in endothelial cell permeability, renal impairment, and mortality, mediated by TRPM7.^16^ Despite this growing body of evidence, the mechanism of TRPM7 in S‐AKI requires investigation.

LPS was utilized to construct an in vitro model of sepsis using RTECs in this study.^17^, ^18^, ^19^ We aimed to elucidate the role of TRPM7 in S‐AKI. A thorough understanding of the occurrence and progression of S‐AKI could provide a theoretical foundation for the prevention and treatment of S‐AKI.

## 2 MATERIALS AND METHODS

### 2.1 Cell culture and cell transfection

Human RTECs (RPTEC/TERT1) were purchased from Genetimes ExCell Technology (CRL‐4031) and cultured in the Dulbecco's modified Eagle's medium (DMEM; Gibco; Thermo Fisher Scientific) supplemented with 5% fetal bovine serum (Gibco) in a humidified atmosphere of 5% CO_2_ at 37°C. RTECs were treated with LPS (10 μg/ml, Macklin) for 12 h^20^ to mimic S‐AKI under in vitro conditions.

Knockdown was performed using small interfering (si)RNAs against TRPM7 (si‐TRPM7#1/2), siRNAs against KLF2 (si‐KLF2#1/2), and nontargeting siRNA as negative control (si‐NC; VectorBuilder). The pcDNA3.1 vector overexpressing KLF2 (pcDNA3.1‐KLF2) and the empty vector (pcDNA3.1) to use as negative control were obtained from Fenghui. Cell transfection was performed using Lipofectamine™ 3000 (Invitrogen), according to the manufacturer's instructions.

### 2.2 Western blot analysis

Total proteins were extracted from RTECs using a radioimmunoprecipitation assay buffer (Biosharp) and quantified by the bicinchoninic acid method (Thermo Fisher Scientific). After denaturing, proteins were separated using 12% sodium dodecyl sulphate polyacrylamide gel electrophoresis. Next, the gel was transferred to polyvinylidene difluoride membranes (Millipore), and membranes were blocked in 5% fat‐free milk for 2 h at room temperature. Membranes were then incubated with specific antibodies at 4°C overnight followed by HRP‐conjugated anti‐rabbit secondary antibody for 1 h at room temperature. Blots were developed using the ECL reagent (Abcam). The gray values were analyzed in Image J v1.46 software (National Institutes of Health). Used antibodies are listed in Table 1.

**Table 1:** Antibodies used for western blot analysis

Antibody	Catalog number	Dilution ratio	Company
Bcl‐2	AB112	1:1000	Beyotime
Bax	AF1270	1:2000	Beyotime
Cleaved‐caspase3	ab32042	1:500	Abcam
Cleaved‐PARP	ab32064	1:5000	Abcam
COX‐2	GTX60935	1:1000	GeneTex
iNOS	GTX17504	1:500	GeneTex
P38 MAPK	GTX110720	1:2000	GeneTex
Phospho‐p38 MAPK	GTX24822	1:1000	GeneTex
p‐ERK1/2	GTX134462	1:1000	GeneTex
ERK1/2	GTX24819	1:1000	GeneTex
p‐JNK	GTX24821	1:500	GeneTex
JNK	GTX52360	1:1000	GeneTex
GAPDH	GTX100118	1:10,000	GeneTex
Goat anti‐rabbit lgG (HRP)	A0208	1:1000	Beyotime

### 2.3 Quantitative real‐time polymerase chain reaction (RT‐qPCR)

Cells were harvested in TRIzol (Invitrogen) and total RNA was converted to cDNA with a High‐Capacity cDNA Reverse Transcription kit (Applied Biosystems). RT‐qPCR was carried out with the QuantiTect SYBR‐Green PCR kit (Qiagen) with pre‐denaturation at 95°C for 30 s, followed by 40 cycles of denaturation at 95°C for 5 s, annealing at 60°C for 30 s, and extension at 72°C for 10 s. The expression of the gene was normalized to that of glyceraldehyde 3‐phosphate dehydrogenase. The primers for reactions are as follows (5ʹ–3ʹ): TRPM7, forward, TGCTTTTGATCTCCTGTCCTGT; reverse, ACAGACAGCCCATATTGCCC; KLF2, forward, GTGGGCATTTTTGGGCTACC; reverse, CCCAGTTCCAAGCAACCAGA; GAPDH, forward, GACTCATGACCACAGTCCATGC; reverse, AGAGGCAGGGATGATGTTCTG.

### 2.4 Cell counting kit‐8 (CCK8) assay

CCK8 assays were used to evaluate the viability of RTECs. In brief, cells (3 × 10^3^ cells/well) were seeded in 96‐well plates and cultured overnight. CCK8 solution (Engreen) was added to each well and the cells were incubated for another 2 h. The optical density (OD) was determined at 450 nm using a microplate reader (Thermo Fisher Scientific).

### 2.5 Determination of lactate dehydrogenase (LDH) and oxidative stress level

The LDH activity, superoxide dismutase (SOD) activity, glutathione peroxidase (GSH‐Px) activity, and malondialdehyde (MDA) level in RTECs were measured using the LDH (ab197004; Abcam), SOD (S0101), GSH‐Px (S0058), and MDA (S0131, Beyotime) assay kit, respectively, according to the manufacturer's instructions.

### 2.6 Terminal deoxynucleotidyl transferase dUTP Nick‐End labeling (TUNEL) assay

TUNEL staining was performed according to the procedure provided in the TUNEL assay kit (Beyotime). Briefly, cells (5 × 10^5^ cells/well) were fixed with 4% paraformaldehyde for 30 min and permeated with phosphate‐buffered saline containing 0.3% Triton X‐100 for another 5 min at room temperature. Following blocking with 3% H_2_O_2_ for 5 min, 4′,6‐diamidino‐2‐phenylindole was used to counterstain the nuclei for 10 min. The results were photographed with a fluorescence microscope (×200 magnification; Olympus Corporation).

### 2.7 Enzyme‐linked immunosorbent assay (ELISA)

The secretion levels of tumor necrosis factor (TNF)‐α (88–7324), interleukin (IL)‐6 (88‐7064), and IL‐1β (88‐7013; Thermo Fisher Scientific) in the RTECs were determined using the specific ELISA kits according to the manufacturer's instructions. Optical density values in each well were measured at a wavelength of 450 nm with a microplate reader.

### 2.8 Luciferase reporter assay

TRPM7 fragments containing the wild‐type (WT) KLF2 binding sites and its mutated form (MUT; ACCTGCGCTAC) were separately inserted downstream of the firefly luciferase gene in the pGL3 vector (Promega), namely TRPM7‐WT and TRPM7‐MUT. For the luciferase reporter assay, cells were cotransfected with pcDNA3.1‐KLF2/pcDNA3.1 and TRPM7‐WT/MUT using Lipofectamine™ 3000. The luciferase activity was determined by a Dual‐Luciferase Reporter Assay System (E1910; Promega) according to the manufacturer's recommendations and normalized to *Renilla* luciferase activity.

### 2.9 Chromatin immunoprecipitation (ChIP) assay

The association between KLF2 and TRPM7 was determined with a ChIP assay kit (Beyotime). Briefly, the cells were fixed with 16% methanol and cross‐linked, which were then treated with a lysis buffer and sonicated. Then, the KLF2 antibody (orb574369; Biorbyt) was added and cultured overnight. Beads were added to harvest the protein–DNA complex. Decrosslinking was then performed with the addition of 5 mmol/L NaCl to retrieve DNA. The enrichment of TRPM7 was examined using RT‐qPCR. The primers for TRPM7 are as follows (5ʹ–3ʹ): forward, CAATTAAGGATAAATCCTGTTCCCC; reverse, TGGCACTTGCCAGAACTCTT.

### 2.10 Bioinformatics analysis

JASPAR (jaspar.genereg.net), an open‐access database of nonredundant transcription factor binding profiles, contains information derived from experimentally confirmed binding sites for transcription factors.^21^ The binding site between the transcription factor KLF2 and the TRPM7 promoter was predicted using the JASPAR database.

### 2.11 Statistical analysis

All experiments were repeated three times independently. Data are expressed as mean ± standard deviation (SD) and statistically analyzed using SPSS 19.0 software (SPSS). Student's *t*‐test and one‐way ANOVA followed by Tukey's post hoc test were applied to evaluate the significance. *p* < .05 was considered to be statistically significant.

## 3 RESULTS

### 3.1 TRPM7 knockdown increases the viability of LPS‐treated cells

**Figure 1:**
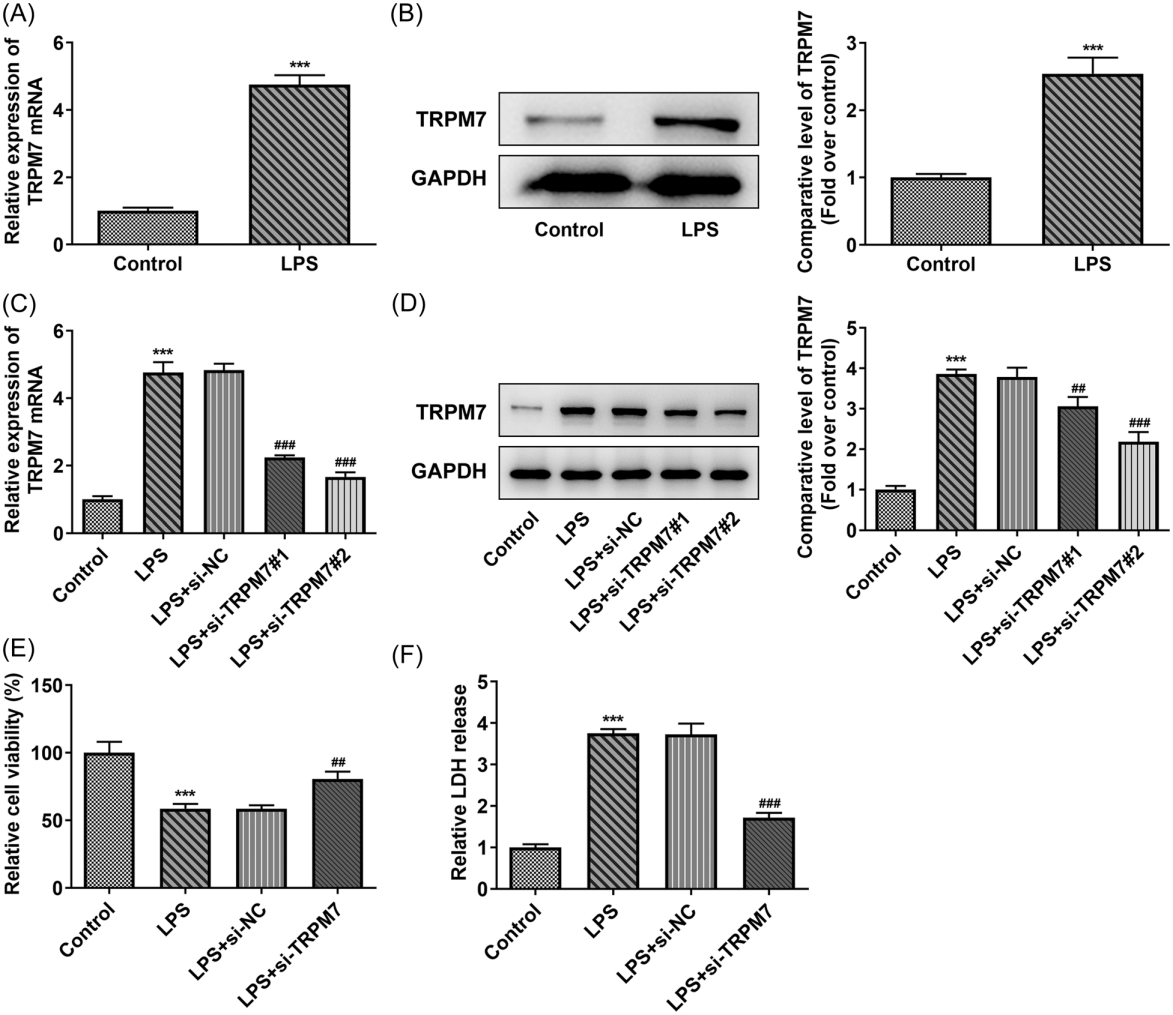
TRPM7 knockdown increases the viability in lipopolysaccharide (LPS)‐treated cells. (A) The level of TRPM7 in the control and LPS‐treated cells was determined using quantitative real‐time polymerase chain reaction (RT‐qPCR) and (B) western blot analysis. (C) The level of TRPM7 in the transfected cells was detected using RT‐qPCR and (D) western blot analysis. (E) The viability in each group was determined using a Cell Counting Kit‐8 (CCK8) assay. (F) The level of lactate dehydrogenase was measured with an assay kit. ***p < .001 versus control; ^##^ *p* < .01, ^###^ *p* < .001 versus LPS + si‐negative control.

The level of TRPM7 in the control and LPS‐treated cells was determined using RT‐qPCR and western blot analysis. The mRNA and protein levels of TRPM7 in the LPS‐treated cells increased compared with that of the control group (Figure 1A,B). TRPM7 expression was knocked down through transfection to investigate its role in RTECs, and the level of TRPM7 in the transfected cells was detected using RT‐qPCR and western blot analysis. The level of TRPM7 in the LPS+si‐TRPM7#1/2 group was lower compared with that of the LPS+si‐NC group. The cells in the LPS+si‐TRPM7#2 group were selected for the subsequent assays due to them displaying a strong downregulation effect (Figure 1C,D). The viability of each group was determined using the CCK8 assay. The viability of the LPS group was decreased, whereas that of the TRPM7 knockdown group partly increased (Figure 1E). Next, the LDH activity was measured with an assay kit. The activity of LDH was elevated in the LPS group and declined in the LPS + si‐TRPM7 group (Figure 1F).

### 3.2 TRPM7 knockdown suppresses apoptosis in LPS‐treated cells

**Figure 2:**
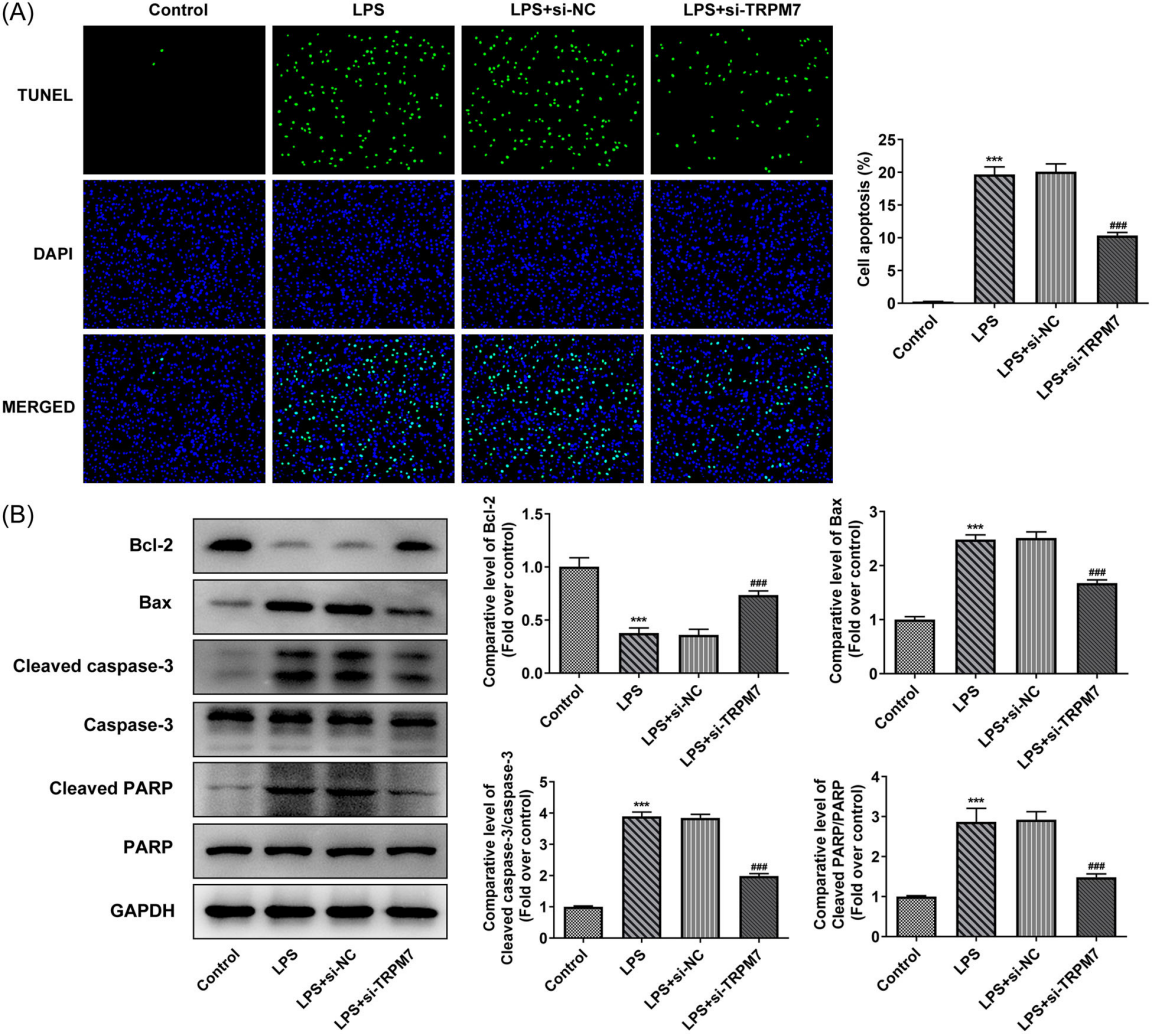
TRPM7 knockdown suppresses apoptosis in lipopolysaccharide (LPS)‐treated cells. (A) Cell apoptosis level in each group was evaluated using Terminal deoxynucleotidyl transferase dUTP Nick‐End Labeling assay. (B) The levels of apoptosis‐related proteins were determined using western blot analysis. ***p < .001 versus control; ^###^ *p* < .001 versus LPS +si‐negative control.

Cell apoptosis in each group was evaluated using TUNEL assay and western blot analysis. The proportion of apoptotic cells in the LPS group was more than those in the control group. TRPM7 knockdown effectively reduced the rate of apoptosis (Figure 2A). Meanwhile, the levels of apoptosis‐related proteins were determined using western blot analysis. The amount of Bcl‐2 was decreased in the LPS group compared to that in the control group, accompanied by an increase in the LPS + si‐TRPM7 group compared with the LPS+si‐NC group. The level of Bax, cleaved caspase 3, and cleaved PARP exhibited a trend opposite to that of Bcl‐2 (Figure 2B).

### 3.3 TRPM7 knockdown reduces the inflammation and oxidative stress in LPS‐treated cells

**Figure 3:**
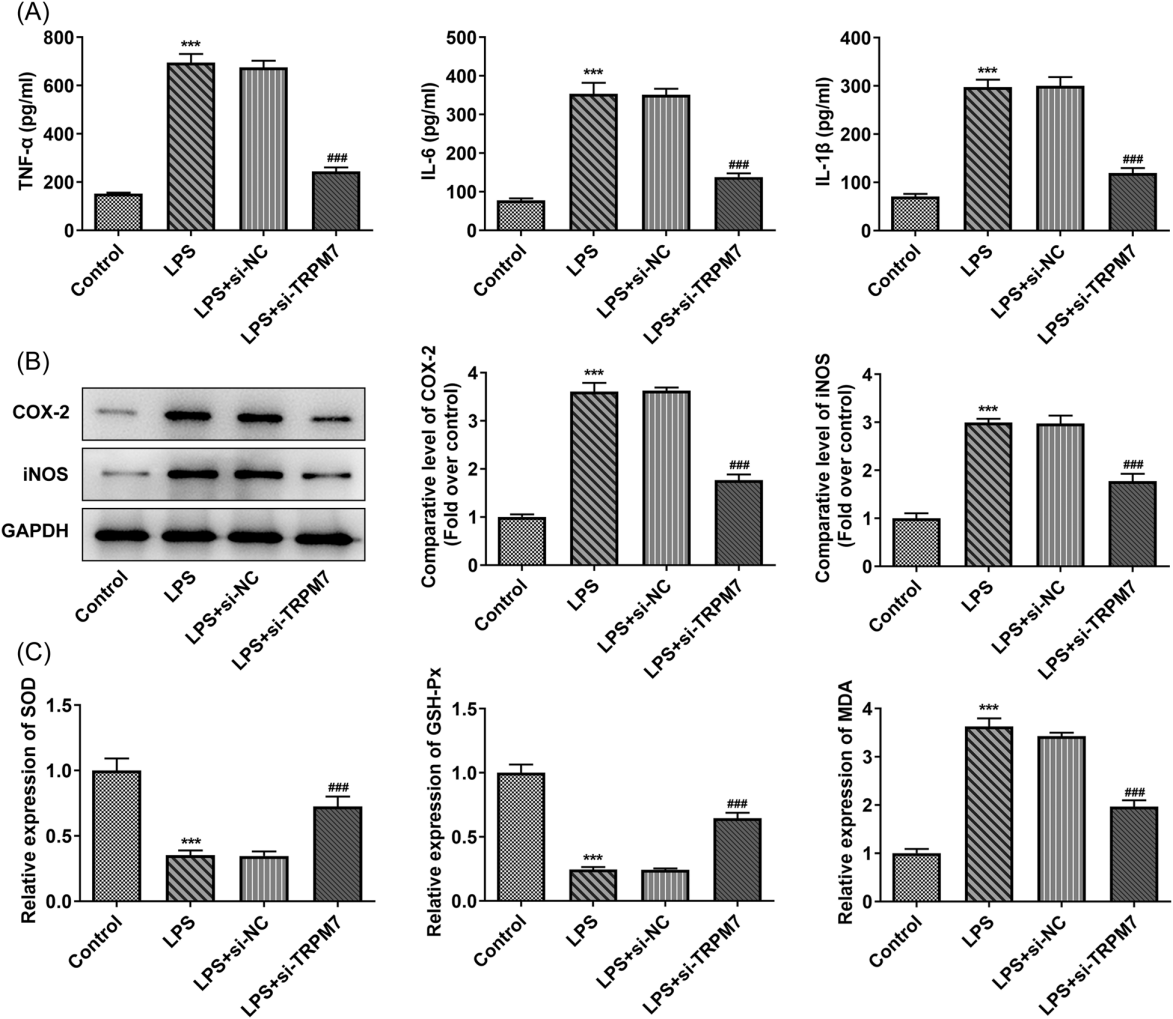
TRPM7 knockdown reduces the inflammation and oxidative stress in lipopolysaccharide (LPS)‐treated cells. (A) The levels of inflammatory factors were determined using an enzyme‐linked immunosorbent assay. (B) The level of cyclooxygenase 2 and inducible nitric oxide synthase were determined using western blot analysis. (C) The oxidative stress level in the cells was measured using assay kits. ***p < .001 versus control; ^##^ *p* < .01, ^###^ *p* < .001 versus LPS+si‐negative control.

The levels of inflammatory factors were determined using ELISA. The levels of TNF‐α, IL‐6, and IL‐1β were elevated in the LPS group and partly declined in the LPS + si‐TRPM7 group (Figure 3A). Furthermore, the cyclooxygenase 2 (COX‐2) and inducible nitric oxide synthase (iNOS) levels were higher in the LPS group and lower in the LPS + si‐TRPM7 group, according to the western blot results (Figure 3B). Afterward, the level of oxidative stress in the cells was determined using assay kits. The activity of SOD and GSH‐Px declined in the LPS group, whereas the level of MDA was elevated. TRPM7 knockdown partially reversed them, as witnessed by the LPS + si‐TRPM7 group (Figure 3C).

### 3.4 KLF2 transcription inhibits TRPM7

**Figure 4:**
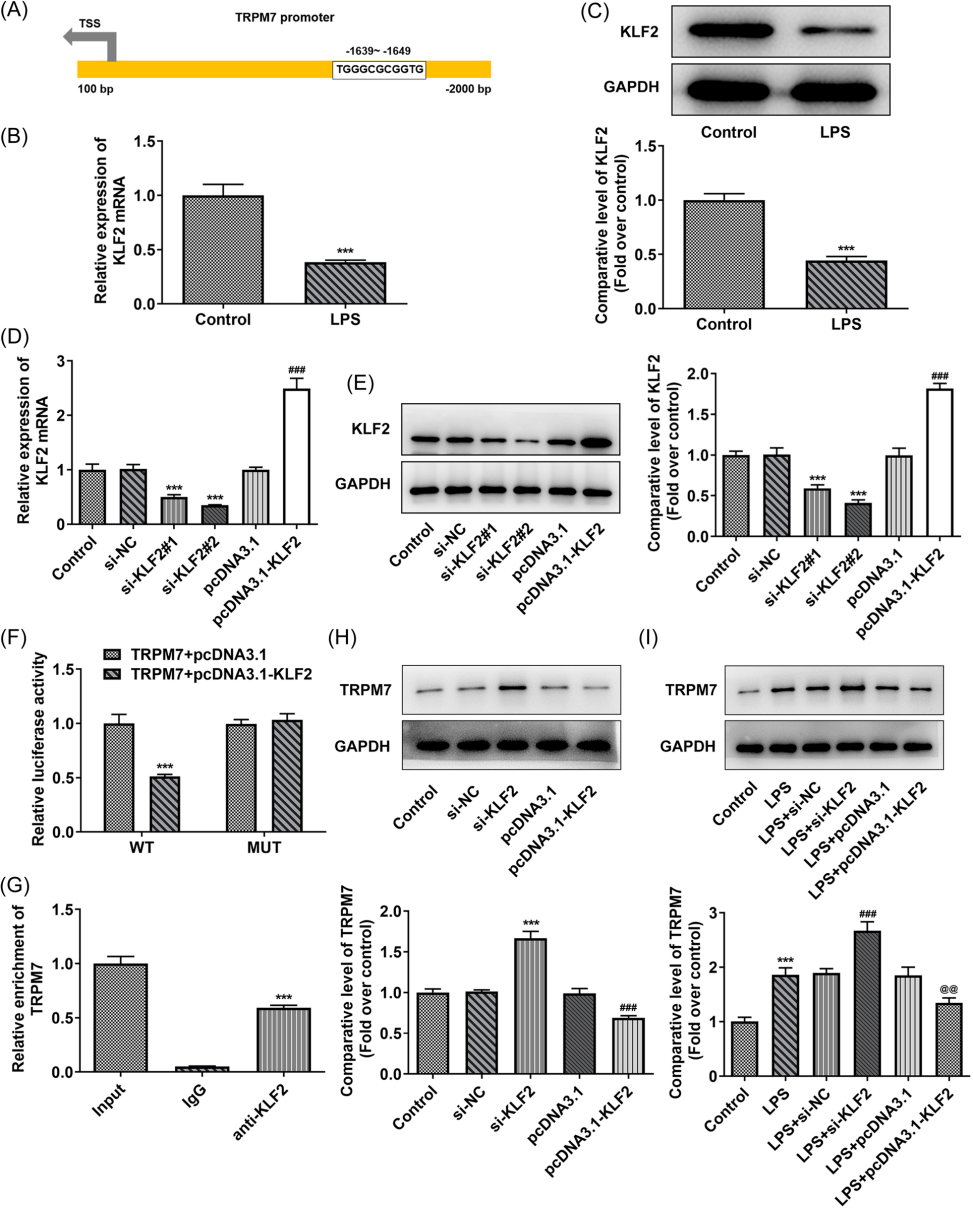
KLF2 transcription inhibits TRPM7. (A) The binding site between Kruppel‐like factor 2 (KLF2) and TRPM7 promoter was predicted in the JASPAR database. (B) The level of KLF2 in the liposaccharide (LPS)‐treated cells was determined using quantitative real‐time polymerase chain reaction (RT‐qPCR) and (C) western blot analysis. ***p < .001 versus control. (D) The transfection efficacy was verified with RT‐qPCR and (E) western blot analysis. ***p < .001 versus si‐NC; ^###^ *p* < .001 versus pcDNA3.1. (F) Promoter activity was detected by a luciferase reporter assay. ***p < .001 versus wild‐type (WT)‐TRPM7+pcDNA3.1. (G) The combining capacity between KLF2 and TRPM7 promotors was assessed using a chromatin immunoprecipitation (ChIP) assay. ***p < .001 versus lgG. (H) The level of TRPM7 in the transfected cells without or (I) with LPS treatment was determined using western blot analysis. ***p < .001 versus control or si‐negative control (NC); ^###^ *p* < .001 versus pcDNA3.1 or LPS + si‐NC; p@@<.01 versus LPS + pcDNA3.1.

The binding location between KLF2 and TRPM7 promoter predicted in the JASPAR database was exhibited in Figure 4A. The level of KLF2 in the LPS‐treated cells was determined using RT‐qPCR and western blot analysis. This level decreased in the LPS‐induced group (Figure 4B,C). Subsequently, cells were transfected individually with si‐KLF2 and pcDNA3.1‐KLF2, and the transfection efficacy was verified using RT‐qPCR and western blot analysis. KLF2 was significantly knocked down in the si‐KLF2#2 group and was strongly expressed in the pcDNA3.1‐KLF2 group (Figure 4D,E). Promoter activity was then detected using a luciferase reporter assay. The relative luciferase activity was markedly declined in the TRPM7‐WT+pcDNA3.1‐KLF2 group compared with that in the TRPM7‐WT+pcDNA3.1 group (Figure 4F). The ChIP assay was used to assess the interaction between KLF2 and the TRPM7 promotor. The result in the anti‐KLF2 group represented the enrichment of TRPM7 (Figure 4G). Following confirmation of KLF2‐TRPM7 binding, the level of TRPM7 in the transfected cells with or without LPS treatment was determined using western blot analysis. In two sets of experiments, KLF2 knockdown elevated the level of TRPM7, whereas KLF2 overexpression reduced the TRPM7 level (Figure 4H). In the LPS treatment group, the level of TRPM7 appeared to be more susceptible compared with KLF2 (Figure 4I).

### 3.5 KLF2 knockdown reverses the effects of TRPM7 knockdown

**Figure 5:**
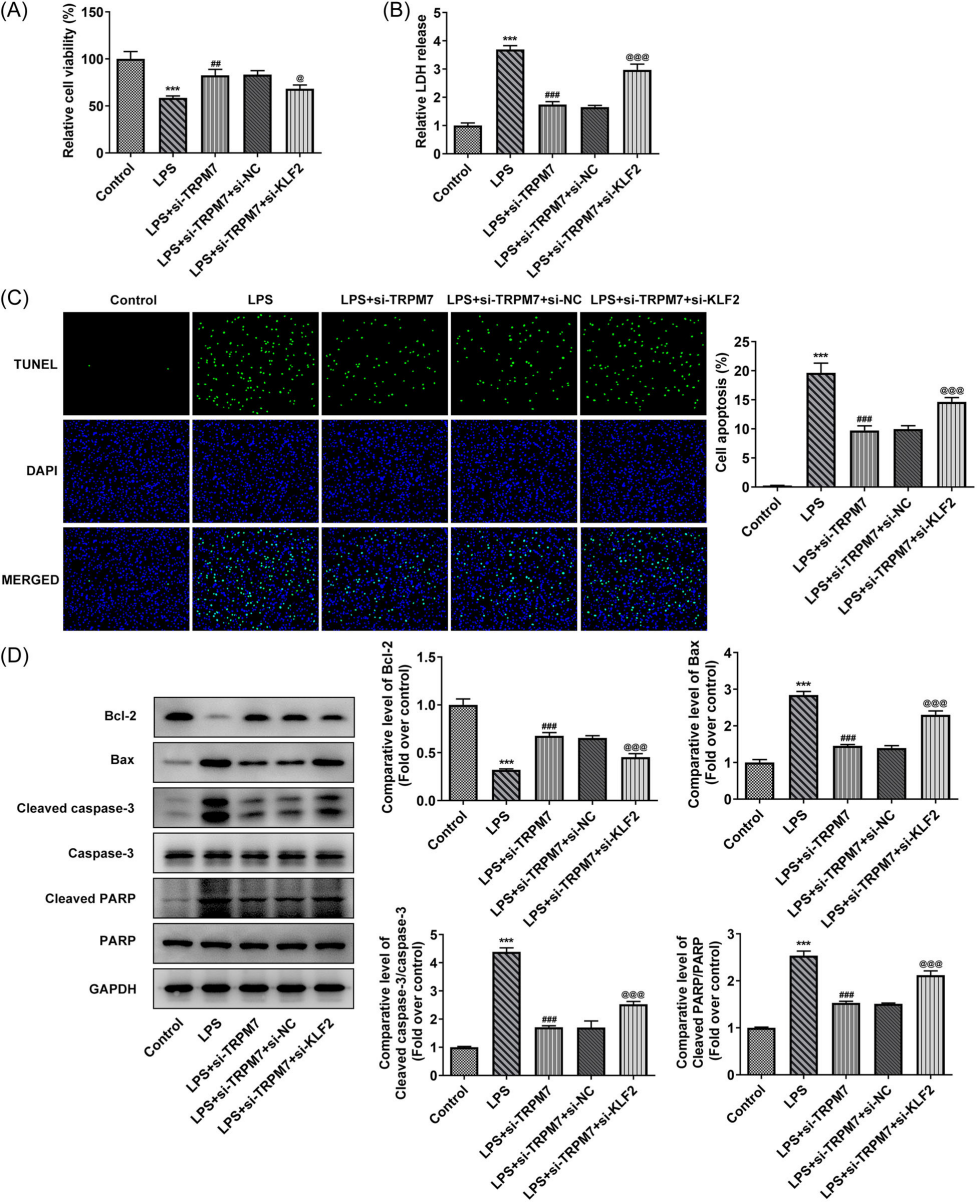
KLF2 knockdown reverses the effects of TRPM7 knockdown on cell viability and apoptosis. (A) The viability in each group was determined using a Cell Counting Kit‐8 assay. (B) The level of lactate dehydrogenase release was measured with an assay kit. (C) Cell apoptosis level in each group was evaluated using terminal deoxynucleotidyl transferase dUTP nick‐end labeling assay. (D) The levels of apoptosis‐related proteins were determined using western blot analysis. ***p < .001 versus control; ^##^ *p* < .01, ^###^ *p* < .001 versus liposaccharide (LPS); ^@^ *p* < .05, p@@@<.001 versus LPS+si‐TRPM7+si‐negative control.

**Figure 6:**
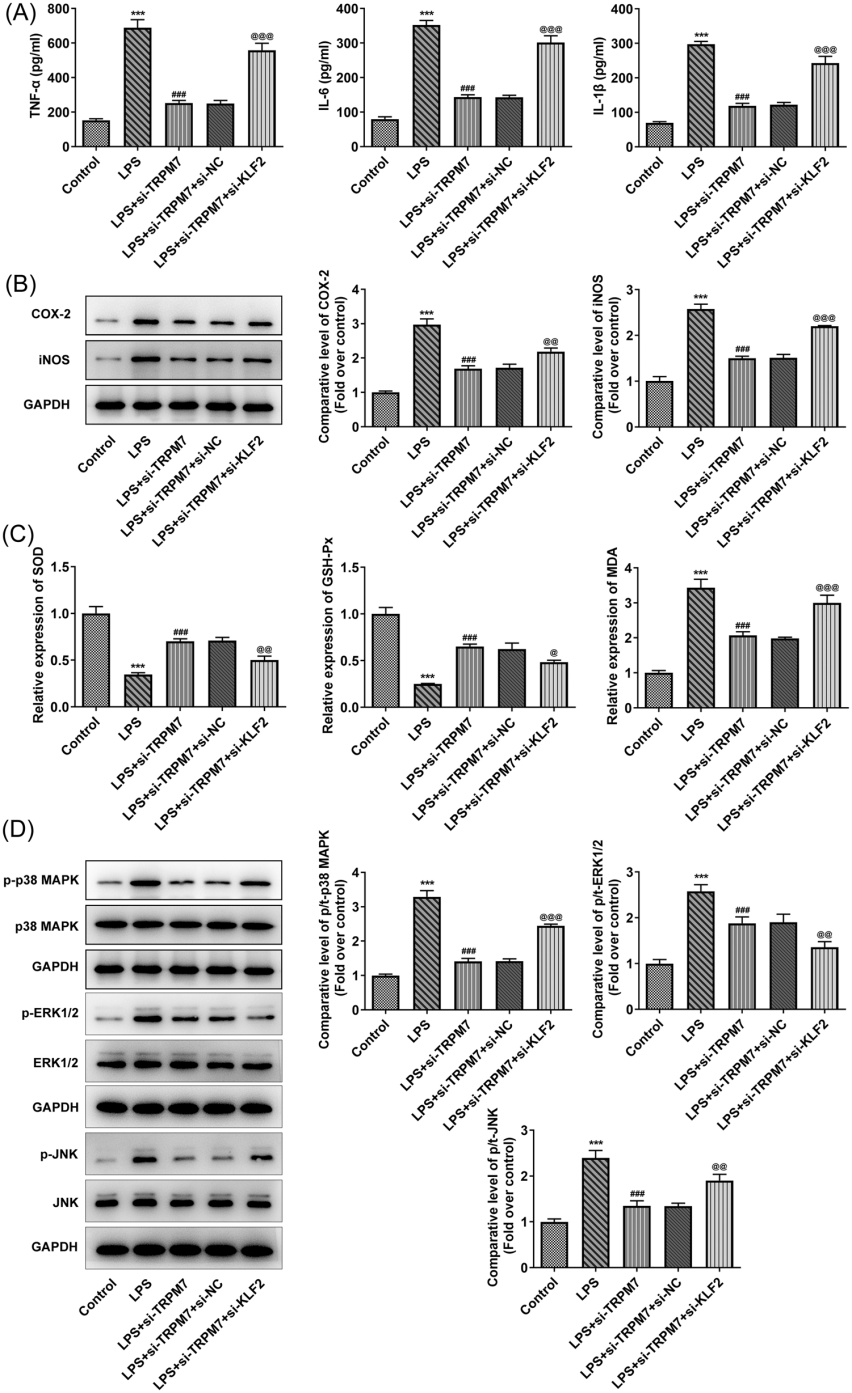
KLF2 knockdown reverses the effects of TRPM7 knockdown on inflammation, oxidative stress, and p38 MAPK signaling. (A) The levels of inflammatory factors were determined using an enzyme‐linked immunosorbent assay (ELISA). (B) The level of cyclooxygenase 2 and inducible nitric oxide synthase (iNOS) were determined using western blot analysis. (C) The oxidative stress level in the cells was measured using assay kits. (D) The abundance of p38 MAPK, ERK1/2, JNK, and their phosphorylated forms in these groups of cells were determined using western blot analysis. ***p < .001 versus control; ^###^ *p* < .001 versus liposaccharide; ^@^ *p* < .05, p@@<.01, p@@@<.001 versus LPS + si‐TRPM7+si‐negative control.

The viability and LDH activity in the above groups of cells was determined. The results revealed that the KLF2 knockdown eliminated the protective effect of TRPM7 knockdown on cell viability (Figure 5A), as well as the inhibition of LDH release (Figure 5B). Cell apoptosis was also assessed using TUNEL assay and western blot analysis. The inhibition of apoptosis rate and decrease in apoptosis‐related protein levels (Bax, cleaved caspase 3, and cleaved PARP) by TRPM7 knockdown was reversed by KLF2 knockdown (Figure 5C,D). Moreover, the influence of KLF2 knockdown on inflammation was determined using ELISA and western blot analysis. The downward trends of TNF‐α, IL‐6, IL‐1β, COX‐2, and iNOS were reversed by KLF2 knockdown (Figure 6A,B). KLF2 knockdown likewise reversed the descending activities of SOD, GSH‐Px, and MDA arising from TRPM7 knockdown (Figure 6C). In addition, the levels of p38 MAPK, ERK1/2, JNK, and their phosphorylated forms in these groups of cells were determined using western blot analysis. TRPM7 knockdown could suppress the phosphorylation of p38 MAPK, ERK1/2, and JNK, whereas additional KLF2 knockdown reversed the inhibited phosphorylation of MAPKs, indicating that KLF2 knockdown activates the MAPK signaling pathway (Figure 6D).

## 4 DISCUSSION

Even though S‐AKI poses a severe medical challenge, it is fortunate that its treatment has progressed due to continual improvements in fluid management, internal environment monitoring, infection control, nutritional support, and renal replacement therapy.^7^, ^22^, ^23^ For a long time, ischemic necrosis was considered to be the fundamental cause of S‐AKI, and inchoate treatment focused on increasing renal blood flow^24^; however, a growing number of investigations have revealed that the pathological alternations of S‐AKI were completely different from nonseptic AKI. In septic shock, blood flow in the renal cortex and medulla is increased rather than decreased, hence, S‐AKI is not caused by ischemia necrosis or ischemia–reperfusion alone.^25^, ^26^ The current consensus is that a complicated interplay between immune mechanisms, activation of inflammatory cascades, and disturbed coagulation pathways contribute to S‐AKI.^26^ Previous rodent studies showed that cell apoptosis in the course of sepsis originates and is localized to the ascending limb of the loop of Henle^27^; however, this has been called into question by subsequent studies. The proximal tubules are more easily injured than the distal tubules, which may be related to the larger protective effects of endocrine and paracrine in the distal tubules, according to a recent study.^28^

RTECs were selected to study the pathology of S‐AKI and found that TRPM7 expression was upregulated in response to stress in LPS‐treated cells. Following TRPM7 knockdown, cell viability increased, LDH activity decreased, and apoptosis was inhibited. Nochy et al. performed renal biopsies from deceased patients with S‐AKI and nonseptic AKI.^29^ Extensive RTEC apoptosis was identified in samples from patients with S‐AKI, but not found in those from patients with nonseptic AKI. This finding indicates that apoptosis of RTECs is particular to patients with S‐AKI, and decreased expression of TRPM7 can inhibit apoptosis. Importantly, this finding suggests that blocking TRPM7 expression could be a promising therapeutic target. Furthermore, as stated in the introduction section, inflammatory mediator release is a high‐risk factor for S‐AKI, and nephroprotection can be achieved by suppressing the cascade of inflammatory factors.^11^ As a scavenger of endogenous oxygen free radicals, SOD can boost vitality and minimize oxidative stress damage in the body.^30^ TRPM7 knockdown has considerable anti‐inflammatory and antioxidative stress effects. It can be concluded that TRPM7 levels can be a crucial indicator of S‐AKI. Nonetheless, its clinical significance must be confirmed through the collection of clinical samples.

Afterward, we investigated the regulatory mechanism of TRPM7. According to JASPAR prediction, the transcription factor KLF2 can bind to the TRPM7 promoter, and its expression was found to be significantly downregulated in LPS‐induced rat alveolar macrophages as previously revealed.^31^ This suggests that upregulation of KLF2 expression may be beneficial for the alleviation of inflammation in S‐AKI. KLF2, widely existing in endothelial cells, is regulated by blood flow shear stress, has an antithrombotic function, and maintains vascular homeostasis.^32^ Herein, KLF2 expression was found to be downregulated in LPS‐treated RTECs, and its level influenced the regulation of TRPM7 in the cells. After being triggered by LPS, the MAPK pathway is activated. Activated p38 MAPK can further phosphorylate and activate several protein kinases and transcription factors, all of which are important in the regulation of inflammatory responses.^33^ TRPM7 knockdown decreased MAPK activation, according to the levels of phosphorylated p38 MAPK, ERK1/2, and JNK protein expression. Given that KLF2 knockdown did not completely reverse the effects of TRPM7 knockdown, we speculate that there might be other transcription factors that regulate TRPM7, which requires further investigation.

To summarize, TRPM7 is negatively controlled by KLF2 transcription and promotes LPS‐induced inflammatory dysfunction via activating the MAPK pathway in RTECs. Our study lays a theoretical foundation for the prevention and management of S‐AKI.

## AUTHOR CONTRIBUTIONS

Yan Sun, Xiaobing Chen, and Xiaomin Li contributed to the conception, design, experiments, and analysis. Yongpeng Xie and Yanli Wang contributed to experiments, acquisition, analysis, and interpretation of data. Qian Zhang and Yu Lu contributed to experiments and analysis. Yan Sun drafted and Xiaomin Li critically revised the article for important intellectual content. All authors have read and approved the final manuscript.

## CONFLICTS OF INTEREST

The authors declare no conflicts of interest.

## Data Availability

The data sets used and/or analyzed during the current study are available from the corresponding author on reasonable request.
